# Standard Model predictions for rare K and B decays without new physics infection

**DOI:** 10.1140/epjc/s10052-023-11222-6

**Published:** 2023-01-24

**Authors:** Andrzej J. Buras

**Affiliations:** 1 TUM Institute for Advanced Study, Lichtenbergstr. 2a, 85747 Garching, Germany; 2 Physik Department, TU München, James-Franck-Straße, 85748 Garching, Germany

## Abstract

The Standard Model (SM) does not contain by definition any new physics (NP) contributions to any observable but contains four CKM parameters which are not predicted by this model. We point out that if these four parameters are determined in a global fit which includes processes that are infected by NP and therefore by sources outside the SM, the resulting so-called SM contributions to rare decay branching ratios cannot be considered as *genuine* SM contributions to the latter. On the other hand *genuine* SM predictions, that are *free* from the CKM dependence, can be obtained for suitable ratios of the *K* and *B* rare decay branching ratios to $\Delta M_s$, $\Delta M_d$ and $|\varepsilon _K|$, all calculated within the SM. These three observables contain by now only small hadronic uncertainties and are already well measured so that rather precise SM predictions for the ratios in question can be obtained. In this context the *rapid test* of NP infection in the $\Delta F=2$ sector is provided by a $|V_{cb}|-\gamma$ plot that involves $\Delta M_s$, $\Delta M_d$, $|\varepsilon _K|$, and the mixing induced CP-asymmetry $S_{\psi K_S}$. As with the present hadronic matrix elements this test turns out to be *negative*, assuming negligible NP infection in the $\Delta F=2$ sector and setting the values of these four observables to the experimental ones, allows to obtain SM predictions for all *K* and *B* rare decay branching ratios that are most accurate to date and as a byproduct to obtain the full CKM matrix on the basis of $\Delta F=2$ transitions *alone.* Using this strategy we obtain SM predictions for 26 branching ratios for rare semileptonic and leptonic *K* and *B* decays with the $\mu ^+\mu ^-$ pair or the $\nu {\bar{\nu }}$ pair in the final state. Most interesting turn out to be the anomalies in the low $q^2$ bin in $B^+\rightarrow K^+\mu ^+\mu ^-$ ($5.1\sigma$) and $B_s\rightarrow \phi \mu ^+\mu ^-$ ($4.8\sigma$).

## Introduction

In this decade and the next decade one expects a very significant progress in measuring the branching ratios for several rare *K* and *B* decays, in particular for the decays $K^+\rightarrow \pi ^+\nu {\bar{\nu }}$, $K_{L}\rightarrow \pi ^0\nu {\bar{\nu }}$, $K_S\rightarrow \mu ^+\mu ^-$, $B_s\rightarrow \mu ^+\mu ^-$, $B_d\rightarrow \mu ^+\mu ^-$, $B\rightarrow K(K^*)\nu {\bar{\nu }}$, $K_L\rightarrow \pi ^0\ell ^+\ell ^-$ [1–3]. Here Belle II, LHCb, NA62, KOTO and later KLEVER at CERN will play very important roles. All these decays are only mildly affected by hadronic uncertainties in contrast to several non-leptonic *B* decays, $K\rightarrow \pi \pi$ decays and in particular the ratio $\varepsilon '/\varepsilon$. As the main hadronic uncertainties for these semi-leptonic and leptonic decays are collected in the formfactors and weak decay constants, further improvements by lattice QCD (LQCD) will reduce these uncertainties to the one percent level. Similar, in the case of the charm contribution to $K^+\rightarrow \pi ^+\nu {\bar{\nu }}$ and $K_L\rightarrow \pi ^0\ell ^+\ell ^-$, long distance effects can be separated from short distance effects and calculated by LQCD. This demonstrates clearly the importance of LQCD calculations [4] in this and coming decades [5]. For *B* physics this is also the case of HQET Sum Rules [6]. But also Chiral Perturbation Theory is useful in this context allowing to extract some non-perturbative quantities from data on the leading Kaon decays [7].

Of particular interest are also semi-leptonic decays $B^+\rightarrow K^+\mu ^+\mu ^-$, $B^0\rightarrow K^{*0}\mu ^+\mu ^-$, $B_s\rightarrow \phi \mu ^+\mu ^-$ and $\Lambda _b\rightarrow \Lambda \mu ^+\mu ^-$ which play an important role in the analyses of the so-called *B*-physics anomalies. They are not as theoretically clean as semi-leptonic decays with neutrinos and in particular leptonic decays but they have the advantage of having larger branching ratios so that several of them have been already measured with respectable precision.

As far as short distance QCD and QED calculations within the Standard Model (SM) of the decay branching ratios in question are concerned, a very significant progress in the last thirty years has been achieved. It is reviewed in [8–11]. In this manner rather precise formulae for SM branching ratios as functions of four CKM parameters [12, 13] can be written down. It will be useful to choose these parameters as follows^1^

1 $$\begin{aligned} \boxed {|V_{us}|,\quad |V_{cb}|, \quad \beta , \quad \gamma } \end{aligned}$$

with $\beta$ and $\gamma$ being two angles in the Unitarity Triangle (UT). Similarly SM expressions for the $\Delta F=2$ observables

2 $$\begin{aligned} \boxed {|\varepsilon _K|,\quad \Delta M_s,\quad \Delta M_d, \quad S_{\psi K_S}}\, \end{aligned}$$

in terms of the CKM parameters can be written down. Due to the impressive progress by LQCD and HQET done in the last decade, the hadronic matrix elements relevant for the latter observables are already known with a high precision. This is even more the case of short distance QCD contributions for which not only NLO QCD corrections are known [15–18] but also the NNLO ones [19–21]and the NLO electroweak corrections [22, 23]. As the experimental precision on $|\varepsilon _K|$, $\Delta M_s$ and $\Delta M_d$ is already impressive and the one on the mixing induced CP-asymmetry $S_{\psi K_S}$, that gives us $\beta$, will be improved by the LHCb and Belle II collaborations soon, this complex of $\Delta F=2$ observables is in a much better shape than $\Delta F=1$ transitions if both the status of the experiment and the status of the theory are simultaneously considered.

We have then a multitude of SM expressions for branching ratios, asymmetries and other observables as functions of only four CKM parameters in (1) that are not predicted in the SM. The remaining parameters like $W^\pm$, $Z^0$, quark and lepton masses and gauge coupling constants or Fermi-constant $G_F$ are already known from other measurements. The question then arises whether not only this system of SM equations describes the existing measurements well, but also what are the SM predictions for rare decay branching ratios measured already for several $b\rightarrow s \mu ^+\mu ^-$ transitions and to be measured for very rare decays with neutrino pair or charged lepton pair in the final state in this and the next decade.

In the 21st century the common practice is to insert all these equations into a computer code like the one used by the CKMfitter [24] and the UTfitter [25] and more recently popular Flavio [26] and HEPfit [27] codes among others. In this manner apparently not only the best values for the CKM parameters can be obtained and consistency checks of the SM predictions can be made. Having the CKM parameters at hand, apparently, one can even find the best SM predictions for various rare decay branching ratios.

While, I fully agree that in this manner a global consistency checks of the SM can be made, in my view the resulting SM predictions cannot be considered as *genuine* SM predictions, simply because the values of the CKM parameters and consequently the Unitarity Triangle, obtained in such a global fit, are likely to depend on possible NP infecting them.^2^ This is in particular the case if some inconsistencies in the SM description of the data for certain observables are found and one has to invoke some models to explain the data. This is in fact the case of several $b\rightarrow s\mu ^+\mu ^-$ transitions for which data are already available.

Moreover there is another problem with such global fits at present. It is the persistent tension between inclusive and exclusive determinations of $|V_{cb}|$ [4, 32]^3^

3 $$\begin{aligned} |V_{cb}|_{\textrm{incl}}= & {} 42.16(50)\times 10^{-3},\nonumber \\ |V_{cb}|_{\textrm{excl}}= & {} 39.21(62)\times 10^{-3}, \end{aligned}$$

which is clearly disturbing because as stressed in [30] the SM predictions for rare decay branching ratios and also $\Delta F=2$ observables in (2) are sensitive functions of $|V_{cb}|$. Therefore the question arises which of these two values should be used in a global fit if any.^4^ As shown recently in [31], the SM predictions for the branching ratios in question and $\Delta F=2$ observables are drastically different for these two values of $|V_{cb}|$. This problem existed already in 2015 in the context of the widely cited paper in [34] as stressed recently in a short note in [28].

But this is not the whole story. Many observables involved in the global fits contain larger hadronic uncertainties than the rare decays listed above and also larger than the $\Delta F=2$ observables in (2) so that SM predictions for theoretically clean decays are polluted in a global fit by these uncertainties. While such observables can be given a low weight in the fit, this uncertainty will not be totally removed.

In my view these are important issues related to global fits that to my knowledge have not been addressed sufficiently in print by anybody. They will surely be important when in the next years the data on a multitude of branching ratios will improve and the hadronic parameters that are not infected by NP will be better known. Therefore, the basic question which I want to address here is whether it is possible to find accurate SM predictions for rare *K* and *B* decays without any NP infection in view of the following three problems which one has to face:

- Several anomalies in semi-leptonic decays, some related to the lepton flavour universality violation.
- Significant tensions between inclusive and exclusive determinations of $|V_{cb}|$ implying very large uncertainties in the SM predictions for rare decay branching ratios and making the use of the values of $|V_{cb}|$ from tree-level decays in this context questionable. Moreover, it is not yet excluded that these tensions are caused by NP [35].
- Hadronic uncertainties in various well measured observables included in a global fit that are often much larger than the ones in rare *K* and *B* decays.

The present paper suggests a possible solution to these problems and studies its implications. It is based on the ideas developed in collaboration with Elena Venturini [30, 31] and extends them in a significant manner. The short note in [28] by the present author, in which some critical comments about the literature have been made, can be considered as an overture to the present paper. In fact our strategy is consistent with the present pattern of experimental data. While significant NP effects have been found in $\Delta F=1$ processes, none in $\Delta F=2$ processes. This peculiar situation has been already addressed in the context of *B* physics anomalies by other authors and we will add a few additional remarks at the end of our paper. However, in none of the related papers in the literature the suggestion has been made to use this fact for the determintation of the CKM parameters without NP infection from $\Delta F=2$ observables alone, so that the strategies developed in [30, 31] and used extensively in the present paper open a new route to phenomenology of flavour violating processes, not only in the SM but also beyond it.

The outline of our paper is as follows. In Sect. 2 we will briefly explain why the SM predictions for rare decays resulting from a global fit cannot be considered as genuine SM predictions unless a careful choice of the observables included in the fit is made. In Sect. 3 I will argue that the strategy developed recently in collaboration with Elena Venturini [30, 31] is presently the most efficient method for obtaining CKM-independent SM predictions for various suitable ratios of rare decay branching ratios to the $\Delta F=2$ observables in (2). In Sect. 4 we address the issue of predicting SM branching ratios themselves. To this end we make the assumption that NP contributions to $\Delta F=2$ observables are negligible which is motivated by a *negative* rapid test that shows a very consistent description of the very precise experimental data on these observables within the SM. This is in addition supported by a new CKM free SM relation (17) between the four $\Delta F=2$ observables in (2) that is in a very good agreement with the data.

Setting the values of $\Delta F=2$ observables to their experimental values and using the CKM-independent ratios found above, allows to obtain SM predictions for all very rare *K* and *B* branching ratios that are most accurate to date [30, 31]. Another bonus of this strategy is the determination of the CKM parameters from $\Delta F=2$ processes *alone*, that allows in turn to make accurate predictions for a number of $|V_{cb}|$-independent ratios that depend on $\beta$ and $\gamma$ [30]. In Sect. 5 using these CKM parameters we find SM predictions for the branching ratios of $B^+\rightarrow K^+\mu ^+\mu ^-$, $B^0\rightarrow K^{*0}\mu ^+\mu ^-$, $B_s\rightarrow \phi \mu ^+\mu ^-$ and $\Lambda _b\rightarrow \Lambda \mu ^+\mu ^-$ and in Sect. 6 SM predictions for several $B_s$ decays with $\nu {\bar{\nu }}$ in the final state are presented.

However, it should be stressed that the predictions in Sects. 5 and  6 go beyond the main strategy of removing CKM parameters from the analyses and in Sect. 7 we repeat the calculation of the decays considered in Sects. 5 and  6 by eliminating $|V_{ts}|$ with the help of $\Delta M_s$ and setting its value to the experimental one. As expected we find very similar results but they are more stable under future modifications of $|V_{ts}|$ due to possible changes in non-perturbative parameters in the $\Delta F=2$ system beyond those relevant for $\Delta M_s$.

**Table 1:** Results for very rare *B* and *K* decay branching ratios using the strategy of [30, 31]. The signs $(\pm )$ in $K_L\rightarrow \pi ^0\ell ^+\ell ^-$ correspond to the constructive and the destructive intereference between directly and indirectly CP-violating contributions. The result for $B^+\rightarrow K^+\nu {\bar{\nu }}$ uses most recent formfactors from HPQCD collaboration [54–56]

Decay	SM branching ratio	Data
$B_s\rightarrow \mu ^+\mu ^-$	$(3.78^{+ 0.15}_{-0.10})\cdot 10^{-9}$	$(3.45\pm 0.29)\cdot 10^{-9}$ [41–44]
$B_d\rightarrow \mu ^+\mu ^-$	${(1.02^{+ 0.05}_{-0.03})}\ \cdot 10^{-10}$	$\le 2.05\cdot 10^{-10}$ [41]
$B_s\rightarrow \tau ^+\tau ^-$	$(7.94^{+ 0.32}_{-0.21})\cdot 10^{-7}$	$\le 6.8\cdot 10^{-3}$ [45]
$B_d\rightarrow \tau ^+\tau ^-$	${(2.14^{+ 0.10}_{-0.06})}\ \cdot 10^{-8}$	$\le 2.1\cdot 10^{-3}$ [45]
$B^+\rightarrow K^+\nu {\bar{\nu }}$	${(5.67\pm 0.32)}\cdot 10^{-6}$	${(11\pm 4)}\cdot 10^{-6}$ [46]
$B^0\rightarrow K^{0*}\nu {\bar{\nu }}$	${(10.13\pm 0.92)}\cdot 10^{-6}$	$\le 1.5\cdot 10^{-5}$ [47]
$B^+\rightarrow \tau ^+\nu _\tau$	$(0.88\pm 0.05)\cdot 10^{-4}$	$(1.06\pm 0.19)\cdot 10^{-4}$ [48]
$B\rightarrow X_s\gamma$	$(3.46\pm 0.24)\cdot 10^{-4}$	$(3.32\pm 0.15)\cdot 10^{-4}$ [48]
$K^+\rightarrow \pi ^+\nu {\bar{\nu }}$	$(8.60\pm 0.42)\cdot 10^{-11}$	$(10.9\pm 3.8)\cdot 10^{-11}$ [49]
$K_{L}\rightarrow \pi ^0\nu {\bar{\nu }}$	$(2.94\pm 0.15)\cdot 10^{-11}$	$\le 3.0\cdot 10^{-9}$ [50]
$(K_S\rightarrow \mu ^+\mu ^-)_{\textrm{SD}}$	$(1.85\pm 0.12)\cdot 10^{-13}$	$\le 2.1\cdot 10^{-10}$ [51]
$K_L\rightarrow \pi ^0e^+e^-(+)$	$(3.48^{+ 0.92}_{-0.80})\cdot 10^{-11}$	$\le 28\cdot 10^{-11}$ [52]
$K_L\rightarrow \pi ^0e^+e^-(-)$	$(1.57^{+ 0.61}_{-0.49})\cdot 10^{-11}$	
$K_L\rightarrow \pi ^0\mu ^+\mu ^-(+)$	$(1.39^{+ 0.27}_{-0.25})\cdot 10^{-11}$	$\le 38\cdot 10^{-11}$ [53]
$K_L\rightarrow \pi ^0\mu ^+\mu ^-(-)$	$(0.95^{+ 0.21}_{-0.20})\cdot 10^{-11}$	

In our view the strategies presented here allow to assess better the pulls in individual branching ratios than it is possible in a global fit, simply because the assumption of the absence of NP is made only in $\Delta F=2$ observables which constitute a subset of observables used in global fits. As within this subset no NP is presently required to describe the data, the resulting SM predictions for rare decays are likely to be free from NP infection. In Sect. 8 we make a few comments on the so-called EXCLUSIVE and HYBRID scenarios based on tree-level decays and considered already in detail in [31]. They could be realized one day if the experts agree on the unique values of $|V_{cb}|$ and $|V_{ub}|$. In Sect. 9 we outline the strategy for finding footprints of NP before one starts using computer codes. A brief summary and an outlook are given in Sect. 10.

Before we start I would like to stress that I am making here a point which I hope will be taken seriously by all flavour practitioners, not only by global fitters. If one does not want to face the tensions in the determination of $|V_{cb}|$ and $|V_{ub}|$ through tree-level decays, the $\Delta F=2$ route is presently the only one possible. The tree-level route explored recently in [29] in detail is presently much harder and is in my view not as transparent as the $\Delta F=2$ route [30, 31, 36] followed here. In particular it did not lead yet to unique values of the CKM parameters because of the tensions between the exclusive and inclusive determinations of $|V_{ub}|$ and $|V_{cb}|$.

In fact the basic idea, beyond the removal of the CKM dependence with the help of suitable ratios [30, 31, 36] and subsequently using *only* $\Delta F=2$ observables to find CKM parameters, can be formulated in a simple manner as follows. Imagine the $\Delta F=2$ archipelago consisting of the four $\Delta F=2$ observables in (2). They can be precisely measured and the relevant hadronic matrix elements can be precisely calculated by using LQCD and HQET Sum Rules. This is sufficient to determine CKM parameters using the SM expressions for these observables finding that this model can consistently describe them [31]. But LQCD and HQET experts can calculate all non-perturbative quantities like weak decay constants, formfactors, hadronic matrix elements etc. so that SM predictions for quantities outside the $\Delta F=2$ archipelago can be made. Comparing these predictions with experiments outside this archipelago one can find out whether there are phenomena that cannot be described by the SM.

**Table 2:** Comparison of the CKM output using the strategy of [30, 31] presented here with UTfitter [70], CKMfitter and PDG22 [58]

CKM	Our fit	UTfitter	CKMfitter	PDG22
$|V_{cb}|\cdot 10^3$	42.6(4)	42.0(5)	$41.5^{+0.4}_{-0.6}$	41.8(8)
$\gamma$	$64.6(16)^\circ$	65.1(13)	$65.5(13)^\circ$	$65.5(15)^\circ$
$|V_{ub}|\cdot 10^3$	3.72(11)	3.71(9)	3.67(8)	3.69(11)
$|V_{ts}|\cdot 10^3$	41.9(4)	41.3(5)	$40.7^{+0.4}_{-0.5}$	41.1(8)
$|V_{td}|\cdot 10^3$	8.66(14)	8.59(12)	$8.52^{+0.08}_{-0.15}$	8.57(20)
${\bar{\varrho }}$	0.164(12)	0.162(10)	$0.157^{+0.009}_{-0.005}$	0.159(10)
${\bar{\eta }}$	0.341(11)	0.347(10)	$0.348^{+0.012}_{-0.005}$	0.348(10)

**Table 3:** SM predictions for $H_1\rightarrow H_2\mu ^+\mu ^-$ branching ratios with $[q^2_{\text {min}},q^2_{\text {max}}]$ compared with the data. Last column gives the pull

Decay	$[q^2_{\text {min}},q^2_{\text {max}}]$	Branching ratio (SM)	Branching ratio (EXP)	Pull
$B^+\rightarrow K^+\mu ^+\mu ^-$	[1.1, 6]	$(2.07\pm 0.16)\cdot 10^{-7}$	$(1.186\pm 0.068)\cdot 10^{-7}$ [88]	$-5.14$
$B^+\rightarrow K^+\mu ^+\mu ^-$	[15, 22]	$(1.26\pm 0.10)\cdot 10^{-7}$	$(0.847\pm 0.050)\cdot 10^{-7}$ [88]	$-3.61$
$B_d^0\rightarrow K^{*0}\mu ^+\mu ^-$	[1.1, 6]	$(2.39\pm 0.28)\cdot 10^{-7}$	$(1.68\pm 0.15)\cdot 10^{-7}$ [89]	$-2.23$
$B_d^0\rightarrow K^{*0}\mu ^+\mu ^-$	[15, 19]	$(2.44\pm 0.26)\cdot 10^{-7}$	$(1.74\pm 0.14)\cdot 10^{-7}$ [89]	$-2.37$
$B_s\rightarrow \phi \mu ^+\mu ^-$	[1.1, 6]	$(2.70\pm 0.25)\cdot 10^{-7}$	$(1.41\pm 0.10)\cdot 10^{-7}$ [90]	$-4.80$
$B_s\rightarrow \phi \mu ^+\mu ^-$	[15, 19]	$(2.28\pm 0.21)\cdot 10^{-7}$	$(1.85\pm 0.13)\cdot 10^{-7}$ [90]	$-1.74$
$\Lambda _b\rightarrow \Lambda \mu ^+\mu ^-$	[1.1, 6]	$(0.53\pm 0.28)\cdot 10^{-7}$	$(0.44\pm 0.31)\cdot 10^{-7}$ [91]	$-0.21$
$\Lambda _b\rightarrow \Lambda \mu ^+\mu ^-$	[15, 20]	$(3.63\pm 0.37)\cdot 10^{-7}$	$(6.00\pm 1.34)\cdot 10^{-7}$ [91]	$+1.70$

**Table 4:** Selective results for SM branching ratios using the strategy of [30, 31] obtained by using the results in [78]. SM1: with our value of $|V_{ts}|$ in (21), SM2: removal of $|V_{ts}|$ using $\Delta M_s$

Decay	SM1	SM2	Data
$B_s\rightarrow \phi \nu {\bar{\nu }}$	$(10.9\pm 0.7)\cdot 10^{-6}$	$(10.9\pm 0.9)\cdot 10^{-6}$	$\le 5.4\cdot 10^{-3}$ [92]
$B_s\rightarrow K^0\nu {\bar{\nu }}$	$(1.4\pm 0.3)\ \cdot 10^{-7}$	$(1.4\pm 0.3)\ \cdot 10^{-7}$	
$B_s\rightarrow K^{0*}\nu {\bar{\nu }}$	$(4.0\pm 0.3)\ \cdot 10^{-7}$	$(4.0\pm 0.4)\ \cdot 10^{-7}$	
$B^0_d\rightarrow X_S\nu {\bar{\nu }}$	$(3.1\pm 0.3)\cdot 10^{-5}$	$(3.1\pm 0.4)\cdot 10^{-5}$	$\le 6.4\cdot 10^{-4}$ [93]
$B^+\rightarrow X_s\nu {\bar{\nu }}$	$(3.3\pm 0.3)\cdot 10^{-5}$	$(3.3\pm 0.4)\cdot 10^{-5}$	$\le 6.4\cdot 10^{-4}$ [93]

To my knowledge there is no analysis in the literature, except for [30, 31], that made SM predictions for rare decay observables using this simple strategy. In the present paper we extend this stategy to several decays not considered in [30, 31], in particular those in which anomalies have been found.

The numerous results following from this strategy are presented in Tables 1, 2, 3 and 4, in the formulae (23)–(36) and (43)–(46). Some of them can be already compared with existing data and many will be compared with improved experimental data which will be available in this decade.

## New physics infected standard model predictions

Let us consider a global SM fit which exposes some deficiencies of this model summarized as anomalies. There are several anomalies in various decays observed in the data, in particular in semi-leptonic *B* decays with a number of branching ratios found below SM predictions, the $(g-2)_\mu$ anomaly and the Cabibbo anomaly among others as reviewed recently in [37]. There is some NP hidden behind these anomalies. The most prominent candidates for this NP are presently the leptoquarks, vector-like quarks and $Z^\prime$. Even if in a SM global fit all these NP contributions are set to zero, in order to see the problematic it is useful to include them in a specific BSM model with the goal to remove these anomalies. The branching ratio for a specific rare decay resulting from such a fit has the general structure

4 $$\begin{aligned} \mathcal {B}(\text {Decay})= \mathcal {B}(\text {Decay})^i_{\text {SM}}+ \mathcal {B}(\text {Decay})^i_{\text {BSM}} \end{aligned}$$

in the case of no intereference between SM and BSM contributions or for decay amplitudes

5 $$\begin{aligned} \mathcal {A}(\text {Decay})= \mathcal {A}(\text {Decay})^i_{\text {SM}}+ \mathcal {A}(\text {Decay})^i_{\text {BSM}} \end{aligned}$$

in the case of the intereferences between SM and NP contributions. The index *i* distinguishes different BSM scenarios. The dependence of the SM part on BSM scenario considered enters exclusively through CKM parameters that in a global fit are affected by NP in a given BSM scenario. Dependently on the BSM scenario, different SM prediction result for a given decay which is at least for me a problem. In the SM there is no NP by definition and there must be a unique SM prediction for a given decay that can be directly compared with experiment.

It could be that for some flavour physicists, who only worked in BSM scenarios and never calculated NLO and NNLO QCD corrections to any decay, this is not a problem. However, for the present author and many of his collaborators as well as other flavour theorists, who spent years calculating higher order QCD corrections to many rare decays, with the goal to find precise genuine SM predictions for various observables, it is a problem and should be a problem. But to me the important question is also whether in a global fit the values in (53) should be taken into account or not. Such questions are avoided in the strategy of [30, 31, 36] because $|V_{cb}|$ is eliminated from the start.

This should also be a problem for LQCD experts who for hadronic matrix elements relevant for $\Delta M_s$, $\Delta M_d$ and $\varepsilon _K$, weak decay constants and formfactors achieved for some of them the accuracy in the ballpark of $1\%$.

In order to exhibit this problematic in explicit terms it is useful to quote the determination of the CKM elements $|V_{cb}|$ and $|V_{ub}|$ from most important flavour changing loop transitions that have been measured, that is meson oscillations and rare *b* hadron decays, including those that show anomalous behaviour [14]

6 $$\begin{aligned} |V_{cb}|_{\text {loop}}= & {} (41.75\pm 0.76)\times 10^{-3}, \nonumber \\ |V_{ub}|_{\text {loop}}= & {} (3.71\pm 0.16)\times 10^{-3}. \end{aligned}$$

The authors of [14] stressed that these values should not be used to obtain SM predictions and we fully agree with them. But in order to assess the size of *B*-physics anomalies properly, we would like to make SM predictions that are not infected by NP. We will soon see that $|V_{cb}|$ in (6) is indeed infected by NP.

It is probably a good place to comment on the very recent paper in [29] in which the authors emphasized that in the process of the determination of the CKM parameters care should be taken to avoid observables that are likely to be affected by NP contributions, in particular the $\Delta F=2$ observables which are key observables for the determination of the CKM parameters in the present paper and also in [30, 31]. Trying to avoid the $\Delta F=2$ observables in their determination of the CKM parameters as much as possible they were forced to consider various scenarios for the $|V_{cb}|$ and $|V_{ub}|$ parameters that suffer from the tensions mentioned above. The fact that exclusive and inclusive values of these parameters imply very different results for rare *K* and *B* decays as well as for the $\Delta F=2$ observables in (2) has been already presented earlier in [31], but the authors of [29] gave additional insights in this problematic. Moreover, they study the issue of the $\gamma$ determinations in non-leptonic *B* decays which will also be important for the tests of our strategy.

Our strategy is much simpler and drastically different from the one of [29] and the common prejudice, also expressed by the latter authors, that $\Delta F=2$ observables are likely to be affected by NP. Presently nobody can claim that these observables are affected by NP. Assuming then, in contrast to [29], that NP contributions to $\Delta F=2$ observables are negligible allows not only to avoid tensions in $|V_{cb}|$ and $|V_{ub}|$ determinations that have important implications on SM predictions for flavour observables [30, 31]. It also allows to determine uniquely and precisely CKM parameters so that various scenarios for them presented in [29] as a result of the tensions in question can be avoided.

Needless to say I find the analysis in [29] interesting and very informative. It will certainly be useful if clear signals of NP will be identified in $\Delta F=2$ observables. Next years will tell us whether their strategy or our strategy is more successful in obtaining SM predictions for a multitude of flavour observables.

## SM predictions for CKM-independent ratios

The only method known to me that allows presently to find SM predictions for rare *K* and *B* decays without any NP infection is to consider suitable ratios of rare decay branching ratios calculated in the SM to the first three $\Delta F=2$ observables in (2), calculated also in the SM, so that the CKM dependence is eliminated as much as possible, in particular the one on $|V_{cb}|$ completely. This proposal in the case of $B_{s,d}\rightarrow \mu ^+\mu ^-$ decays, that in fact works for all *B*-decays governed by $|V_{td}|$ and $|V_{ts}|$ couplings, goes back to 2003 in which the following CKM-independent SM ratios have been proposed [36]

7 $$\begin{aligned} \boxed {\begin{aligned}R_q&=\frac{\overline{\mathcal {B}}(B_q\rightarrow \mu ^+\mu ^-)}{\Delta M_q}\\&= 4.291\times 10^{-10}\ \frac{\tau _{B_q}}{{\hat{B}}_q}\frac{(Y_0(x_t))^2}{S_0(x_t)},\quad q=d,s , \end{aligned}} \end{aligned}$$

with $Y_0$ and $S_0$ known one loop $m_t$-dependent functions. The parameters ${\hat{B}}_q$ are known already with good precision from LQCD [38]. The “bar” on the branching ratios takes into account the $\Delta \Gamma _q$ effects that are only relevant for $B_s\rightarrow \mu ^+\mu ^-$ [39].

Recently this method has been generalized to rare Kaon decays. Presently the most interesting $|V_{cb}|$-independent ratios in this case read [30, 31]^5^

8 $$\begin{aligned}{} & {} \boxed {\begin{aligned} R_{11}(\beta ,\gamma )&=\frac{\mathcal {B}(K^+\rightarrow \pi ^+\nu {\bar{\nu }})}{|\varepsilon _K|^{0.82}}\\&=(1.31\pm 0.05)\times 10^{-8}\left( \frac{\sin \gamma }{\sin 67^\circ }\right) ^{0.015}\\&\quad \left( \frac{\sin 22.2^\circ }{\sin \beta }\right) ^{0.71}, \end{aligned}} \end{aligned}$$

9 $$\begin{aligned}{} & {} \boxed {\begin{aligned}R_{12}(\beta ,\gamma )&=\frac{{\mathcal {B}}(K_{L}\rightarrow \pi ^0\nu {\bar{\nu }})}{|\varepsilon _K|^{1.18}}\\&=(3.87\pm 0.06)\times 10^{-8} \left( \frac{\sin \gamma }{\sin 67^\circ }\right) ^{0.03}\\&\quad \left( \frac{\sin \beta }{\sin 22.2^\circ }\right) ^{0.9{8},}\end{aligned}} \end{aligned}$$

where the ratios $R_q$, $R_{11}$ and $R_{12}$ belong to the set of 16 $|V_{cb}|$-independent ratios proposed in [30]. We will encounter them in Sect. 4.4.

It should be stressed that these ratios are valid *only* within the SM. It should also be noted that the only relevant CKM parameter in these $|V_{cb}|$-independent ratios is the UT angle $\beta$ and this is the reason why we need the mixing induced CP-asymmetry $S_{\psi K_S}$ to obtain predictions for $K^+\rightarrow \pi ^+\nu {\bar{\nu }}$ and $K_{L}\rightarrow \pi ^0\nu {\bar{\nu }}$. While $\gamma$ also enters these expressions, its impact on final results is practically irrelevant. This is still another advantage of this strategy over global fits in addition to the independence of $|V_{cb}|$ because while $\beta$ is already rather precisely known, this is not the case for $\gamma$:

10 $$\begin{aligned} \boxed {\beta =(22.2\pm 0.7)^\circ , \quad \gamma = (63.8^{+3.5}_{-3.7})^\circ .} \end{aligned}$$

Here the value for $\gamma$ is the most recent one from the LHCb which updates the one in [40] $(65.4^{+3.8}_{-4.2})^\circ$. However, as we will see below our strategy will allow the determination of $\gamma$ that is significantly more precise than this one and in full agreement with the LHCb value above.

Yet, even if in the coming years the determination of $\gamma$ by the LHCb and Belle II collaboratios will be significantly improved and this will certainly have an impact on global fits, this will have practically no impact on the SM predictions for the four ratios listed above. On the other hand the improvement on the measurement of $\beta$ will play more important role for $R_{11}$ and $R_{12}$ and thereby also for $K^+\rightarrow \pi ^+\nu {\bar{\nu }}$ and $K_{L}\rightarrow \pi ^0\nu {\bar{\nu }}$ decreasing the uncertainty in the SM predictions for both decays. For $K^+\rightarrow \pi ^+\nu {\bar{\nu }}$ further improvement will be obtained by reducing the uncertainty in long distance charm contribution through LQCD computations [5]. Then the uncertainty in the numerical factor in $R_{11}$ will be further decreased allowing to test the SM in an impressive manner when the $K^+\rightarrow \pi ^+\nu {\bar{\nu }}$ branching ratio will be measured at CERN in this decade with an accuracy of $5\%$.

Before continuing let us stress again that the results for the ratios $R_q$, $R_{11}$ and $R_{12}$ are only valid in the SM and being practically independent of the CKM parameters can be regarded as genuine SM predictions for the ratios in question. Except for $\beta$ obtained using SM expression

11 $$\begin{aligned} S_{\psi K_S}=\sin (2\beta )=0.699(17)\, \end{aligned}$$

I do not have to know other CKM parameters to obtain the SM predictions listed above.

The experimental values of the $\Delta F=2$ observables in (2) are already known with high precision. Once the four branching ratios will be experimentally known these four ratios will allow a very good test of the SM without any knowledge of the CKM parameters except for $\beta$ in the case of $K^+\rightarrow \pi ^+\nu {\bar{\nu }}$ and $K_{L}\rightarrow \pi ^0\nu {\bar{\nu }}$. We will return to other ratios in Sect. 4.4.

## SM predictions for rare decay branching ratios

### Main strategy and first results

But this is the story of the ratios. We would like to make one step further and obtain SM predictions for branching ratios themselves. The proposal of [30, 31] is to use in the ratios in question the experimental values for the $\Delta F=2$ observables in (2) to predict the branching ratios for rare *K* and *B* decays. There are four arguments for this procedure:

- The experimental status of $\Delta F=2$ observables is much better than the one of rare decays and their theoretical status is very good.
- To obtain SM predictions for branching ratios that are not infected by NP the only logical possibility is to assume that SM describes properly $\Delta F=2$ observables not allowing them to be infected by NP.
- The latter assumption is supported by the data on $\Delta F=2$ observables as pointed out in [31] and repeated below. There is presently no need for NP contributions to $\Delta F=2$ observables to fit the data.
- There is no other sector of flavour observables that can determine all CKM parameters beyond $|V_{us}|$, in particular $|V_{ub}|$ and $|V_{cb}|$, in which the tensions between inclusive and exclusive determinations of the latter can be avoided.

Inserting then experimental values of $\Delta M_q$ into (7) and using the most recent LQCD values of ${\hat{B}}_q$ from [38], as listed in Table 5, one finds the results for $B_{s,d}\rightarrow \mu ^+\mu ^-$ and the remaining rare *B* decays in Table 1. Similar, setting the experimental value of $|\varepsilon _K|$ into (8) and (9) and including all theoretical uncertainties and experimental ones from $|\varepsilon _K|$ and $\beta$ in (10) one finds the results for $K^+\rightarrow \pi ^+\nu {\bar{\nu }}$ and $K_{L}\rightarrow \pi ^0\nu {\bar{\nu }}$ and subsequently for the remaining rare *K* decays in Table 1.

These are the most precise SM predictions for decays in question to date. In particular in the case of $K\rightarrow \pi \nu {\bar{\nu }}$ they supersede the widely cited 2015 results [34]

12 $$\begin{aligned}{} & {} {\mathcal {B}}(K^+\rightarrow \pi ^+\nu {\bar{\nu }})_\text {SM}= (8.4\pm 1.0)\times 10^{-11} , \nonumber \\{} & {} {\mathcal {B}}(K_{L}\rightarrow \pi ^0\nu {\bar{\nu }})_\text {SM}=(3.4\pm 0.6)\times 10^{-11}, \quad (2015),\nonumber \\ \end{aligned}$$

that are clearly out of date as stressed recently in a note by the author [28]. Using our strategy the uncertainties in the two branching ratios have been reduced by a factor of 2.4 and 4.0, respectively.

Relative to [30, 31] the predictions for $K_L\rightarrow \pi ^0\ell ^+\ell ^-$ are new. Moreover, we added to the error in the prediction for $(K_S\rightarrow \mu ^+\mu ^-)_{\textrm{SD}}$ the uncertainty from the indirect CP violation pointed out recently in [57]. Adding it in quadrature the error has been increased from 5.4 to $6.5\%$. Our final result differs from the one of these authors because for the CKM parameters they use the UT fit from PDG22 [58] that differs from our strategy. See Sect. 4.3 for more details.

Among the results shown in Table 1 the most interesting until recently was a $2.7\sigma$ anomaly in $B_s\rightarrow \mu ^+\mu ^-$, but according to the most recent messages from CMS and HFLAV this branching ratio has been increased to $3.45(29)\cdot 10^{-9}$ as given in Table 1 thereby eliminating this anomaly. In this context I would like to comment on the widely cited by experimentalists SM prediction from [59] $3.66(12)\cdot 10^{-9}$. It is based on NLO QCD [60–63], NNLO QCD [64], NLO electroweak [65] and QED corrections calculated in [59]. However, it does not properly represent the SM value because the inclusive value of $|V_{cb}|$ has been used to obtain it. As shown in [31], for the exclusive value of $|V_{cb}|$ one finds $3.18(12)\cdot 10^{-9}$. Interestingly the CMS2022 result alone with $3.83(42)\cdot 10^{-9}$ agrees perfectly with our $|V_{cb}|$ independent result in Table 1 which is based on all the perturbative calculations listed above but uses (7) to eliminate $|V_{cb}|$. In fact our SM prediction has been obtained several months before the new CMS value [31].

The recent result on $B^+\rightarrow K^+\nu {\bar{\nu }}$ from Belle II with data visibly *above* the SM prediction is also interesting but the experimental error is still large. We are looking forward to the final CMS and Belle II analyses and the corresponding ones from LHCb and ATLAS so that more precise values on both branching ratios will be available from HFLAV.

In this context a number of important comments should be made. This method for obtaining precise SM predictions has been questioned by a few flavour researchers who claim the superiority of global fits in obtaining SM predictions over the novel methods developed in [30, 31, 36] that allowed to remove the sensitivity of SM predictions not only to $|V_{cb}|$ but also to $\gamma$. The criticism is related to the second item in our proposal, namely the use of the experimental values for $\Delta F=2$ observables in this strategy, with the goal to obtain SM predictions. The claim is that the presence of NP in the $\Delta F=2$ observables would invalid the full procedure.

In my view, that is supported by a number of my colleagues, this criticism misses the following important point. The only assumption made in our procedure is that $\Delta F=2$ observables in (2) are not infected by NP. In a global fit this assumption is made for many additional observables and the chance of an infection is much larger. One should also stress that the formulae used to obtain the four ratios in (7)–(9), are only valid in the SM and in the SM world there are no NP contributions. Therefore, if one wants to obtain *genuine* SM predictions for rare decay branching ratios using these ratios, it is simply mandatory to set, in the formulae (7)–(9), the quantities in (2) to their experimental values. If one day it will turn out that NP infects $\Delta F=2$ processes, then anyway one will have to repeat the full analysis in a NP model that will result in predictions for rare decays in this particular model, not in the SM.

One can also give a simpler argument for the validity of this strategy. Formulae (7)–(9) represent SM correlations between chosen $\Delta F=1$ branching ratios and the $\Delta F=2$ observables in question. Setting $\Delta M_s$ to its experimental value gives automatically the SM prediction for $B_s\rightarrow \mu ^+\mu ^-$ and similarly for the other three branching ratios. Note that in the case of (8) and (9) these are not just correlations between $K^+\rightarrow \pi ^+\nu {\bar{\nu }}$ and $K_{L}\rightarrow \pi ^0\nu {\bar{\nu }}$ branching ratios and $|\varepsilon _K|$ but with the latter raised to appropiate power so that $|V_{cb}|$ and $\gamma$ dependences are eliminated.

**Fig. 1:**
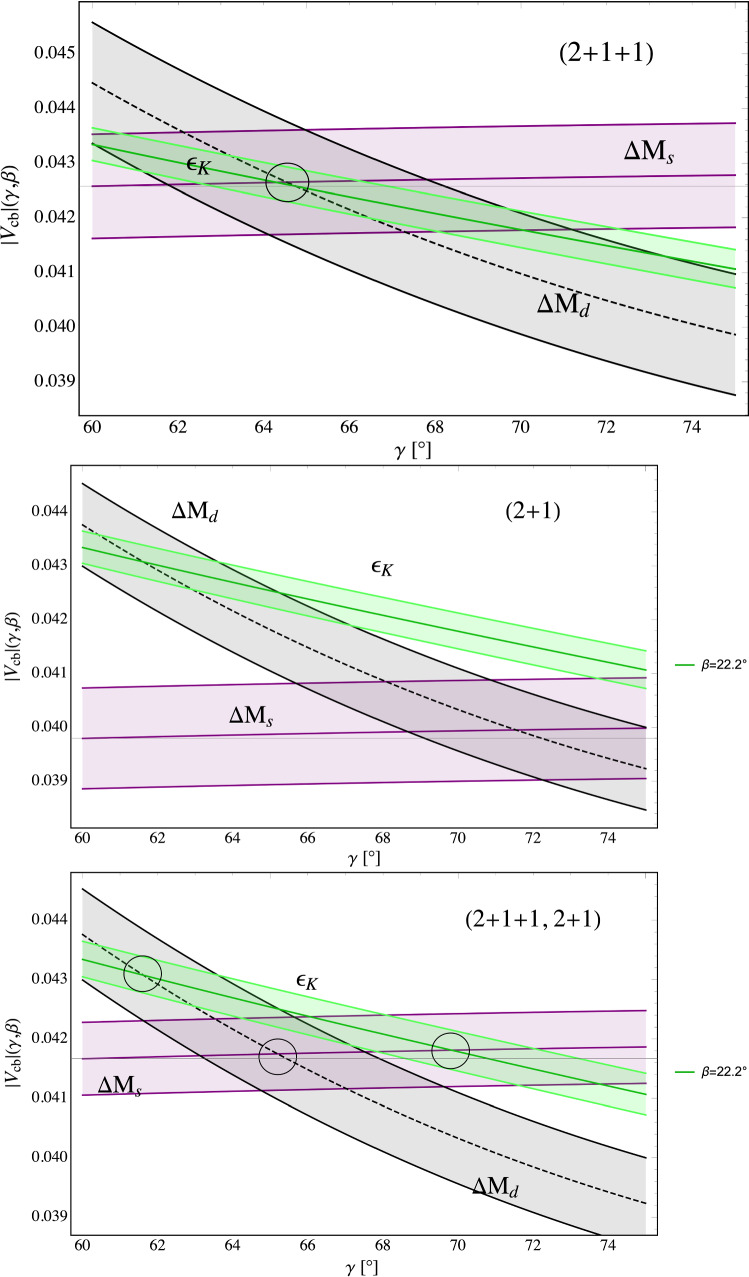
Three rapid tests of NP infection in the $\Delta F=2$ sector taken from [31] as explained in the text. The values of $|V_{cb}|$ extracted from $\varepsilon _K$, $\Delta M_d$ and $\Delta M_s$ as functions of $\gamma$. $2+1+1$ flavours (top), $2+1$ flavours (middle), average of $2+1+1$ and $2+1$ cases (bottom). The green band represents experimental $S_{\psi K_S}$ constraint on $\beta$

I do hope very much that this underlines again the important role of correlations between various observables, not only within the SM but also in any model as discussed at length in [10, 66, 67]. In my view before doing any global fit it is useful to find first these correlations and compare them with data. Within the SM they allow to reduce the dependence on the CKM parameters to the minimum.

### Rapid test for the $\Delta F=2$ sector

Having set the SM expressions for $\Delta F=2$ observables to their experimental values we are now in the position to determine the CKM parameters. However, before doing it, it is mandatory to perform a rapid test to be sure that the resulting CKM parameters are not infected by NP. To this end, instead of inserting the formulae in a computer program right away it is useful to construct first a $|V_{cb}|-\gamma$ plot [30, 31] with three bands resulting separately from $\Delta M_s$, $\Delta M_d$ and $|\varepsilon _K|$ constraint and in the latter case imposing the constraint from $S_{\psi K_S}$. The superiority of the $|V_{cb}|-\gamma$ plot with respect to $|V_{cb}|$ and $\gamma$ over UT plots has been recently emphasized in [68].

The plots in Fig. 1, taken from [31], illustrate three *rapid tests* of NP infection of the $\Delta F=2$ sector. The test is *negative* if these three bands cross each other at a small common area in this plane so that unique values of $|V_{cb}|$ and $\gamma$ are found. Otherwise it is *positive* signalling NP infection. Indeed, as seen in the first $|V_{cb}|-\gamma$ plot in Fig. 1 that is based on $2+1+1$ LQCD hadronic matrix elements [38], the SM $|V_{cb}|-\gamma$ bands resulting from $\varepsilon _K$, $\Delta M_d$ and $\Delta M_s$ after imposition of the $S_{\psi K_S}$ constraint, turn out to provide such unique values of $|V_{cb}|$ and $\gamma$. No sign of NP infection in this case. On the other hand, as seen in the remaining two plots in Fig. 1, this is not the case if $2+1$ or the average of $2+1+1$ and $2+1$ hadronic matrix elements LQCD are used. In these two cases the test turns out to be *positive*.

Explicitly these three bands in the $2+1+1$ case are represented by the expressions [68]

$$\underline{|\varvec{\varepsilon }_\textbf{K}|}$$

13 $$\begin{aligned} |V_{cb}|= & {} 42.6\times 10^{-3}\,\left[ \frac{\sin (64.6^\circ )}{\sin \gamma } \right] ^{0.491} \left[ \frac{\sin (\beta )}{\sin (22.2^\circ )} \right] ^{0.256} \nonumber \\{} & {} \times \left[ \frac{0.7625}{{\hat{B}}_K}\right] ^{0.294} \left[ \frac{|\varepsilon _K|}{2.224\times 10^{-3}} \right] ^{0.294} , \end{aligned}$$

$$\underline{\varvec{\Delta } {\textbf{M}}_{\textbf{d}}}$$

14 $$\begin{aligned} |V_{cb}|= & {} 42.6\times 10^{-3}\,\left[ \frac{\sin (64.6^\circ )}{\sin \gamma } \right] \left[ \frac{210.6\text {MeV}}{\sqrt{{\hat{B}}_{B_d}}F_{B_d}}\right] \nonumber \\{} & {} \times \left[ \frac{2.307}{S_0(x_t)}\right] ^{0.5} \left[ \frac{0.5521}{\eta _B}\right] ^{0.5}\, \left[ \frac{\Delta M_d}{0.5065/\textrm{ps}} \right] ^{0.5} , \end{aligned}$$

$$\underline{\varvec{\Delta } {\textbf{M}}_{\textbf{s}}}$$

15 $$\begin{aligned} |V_{cb}|= & {} \left[ \frac{41.9\times 10^{-3}}{G(\beta ,\gamma )}\right] \left[ \frac{256.1\text {MeV}}{\sqrt{{\hat{B}}_{B_s}}F_{B_s}}\right] \left[ \frac{2.307}{S_0(x_t)}\right] ^{0.5}\nonumber \\{} & {} \times \left[ \frac{0.5521}{\eta _B}\right] ^{0.5}\, \left[ \frac{\Delta M_s}{17.749/\textrm{ps}} \right] ^{0.5}\,~~~~~~~~ \end{aligned}$$

with $\hat{B}_K =0.7625(97)$ [4] and the remaining parameters given in Table 5. Moreover,

16 $$\begin{aligned} G(\beta ,\gamma )= 1 +\frac{\lambda ^2}{2}(1-2 \sin \gamma \cos \beta ) . \end{aligned}$$

Further details on these formulae can be found in [30, 31, 68].

Consequently, with the presently known values of the non-perturbative parameters from LQCD in Table 5 and the experimental value of $\beta$, the SM is performing in the $\Delta F=2$ sector very well. No NP is required in this sector to describe the data. This test will improve with the reduction of the uncertainties in ${\hat{B}}_K$, $\sqrt{{\hat{B}}_{B_d}}F_{B_d}$, $\sqrt{{\hat{B}}_{B_s}}F_{B_s}$ and $\beta$. Therefore it is very important that several LQCD collaborations perform simulations with 2+1+1 flavours.

All this can also be seen with the help of the following, practically CKM free, SM relation between the four $\Delta F=2$ observables in (2) which we present here for the first time. It reads

17 $$\begin{aligned} \boxed {\begin{aligned}\frac{ |\varepsilon _K|^{1.18}}{\Delta M_d\,\Delta M_s}&=(8.22\pm 0.18)\times 10^{-5}\, \\&\quad \left( \frac{\sin \beta }{\sin 22.2^\circ }\right) ^{1.027} K\,\mathrm{ps^2},\end{aligned}} \end{aligned}$$

where

18 $$\begin{aligned} K= & {} \left( \frac{{\hat{B}}_K}{0.7625}\right) ^{1.18} \left[ \frac{210.6\text {MeV}}{\sqrt{{\hat{B}}_{B_d}}F_{B_d}}\right] ^2\, \left[ \frac{256.1\text {MeV}}{\sqrt{{\hat{B}}_{B_s}}F_{B_s}}\right] ^2\,\nonumber \\= & {} \, 1.00\pm 0.07 . \end{aligned}$$

Similar to the relations (8) and (9) the dependence on $|V_{cb}|$ drops out and the one on $\gamma$ being negligible is included in the uncertainty varying $\gamma$ in the range $60^\circ \le \gamma \le 70^\circ$. Inserting the experimental values of the three $\Delta F=2$ observables on the l.h.s one finds for this ratio $(8.26\pm 0.06)\times 10^{-5}$. Consequently, with the presently known values of the non-perturbative parameters from LQCD in Table 5 and the present value of $\beta$ from $S_{\psi K_S}$, the SM is performing in the $\Delta F=2$ sector indeed very well. However with the $2+1$ flavours the central value on the r.h.s of (17) decreases to $(6.29\pm 0.18)\times 10^{-5}$ so that the fact that this ratio agrees with the data for present values of hadronic parameters with $2+1+1$ flavours and the experimental value of $\beta$ is remarkable.

What if the rapid test turns out to be *positive* one day. Then it is safer to just compare the SM predictions for the ratios of branching ratios like the ones in (7)–(9) which being independent of CKM parameters are valid in the SM independently of NP present in $\Delta F=2$ processes. In this case the restriction of the fit of the CKM parameters to $\Delta F=2$ processes is mainly motivated by the desire to avoid the involvement of the tensions between different determinations of $|V_{cb}|$ and $|V_{ub}|$. However, with the present accuracy of the hadronic parameters the present rapid test is clearly negative.

It is possible that one can determine CKM parameters by increasing the number of observables beyond $\Delta F=2$ observables used by us, but then it should be an obligation to perform a rapid test using $|V_{cb}|-\gamma$ plot that includes additional observables before one could claim that the resulting SM predictions for rare branching ratios are indeed genuine SM predictions.

### CKM parameters

#### Our determination

The determination of $\gamma$ and $|V_{cb}|$ can be further improved by considering first the $|V_{cb}|$-independent ratio $\Delta M_d/\Delta M_s$ from which one derives an accurate formula for $\sin \gamma$

19 $$\begin{aligned} \sin \gamma= & {} \frac{0.983(1)}{\lambda }\sqrt{\frac{m_{B_s}}{m_{B_d}}}\xi \sqrt{\frac{\Delta M_d}{\Delta M_s}} , \nonumber \\ \xi= & {} \frac{\sqrt{{\hat{B}}_{B_s}}F_{B_s}}{\sqrt{{\hat{B}}_{B_d}}F_{B_d}}=1.216(16), \end{aligned}$$

with the value for $\xi$ from [38]. The advantage of using this ratio over studying $\Delta M_s$ and $\Delta M_d$ separately is its $|V_{cb}|$-independence and the reduced error on $\xi$ from LQCD relative to the individual errors on hadronic parameters in $\Delta M_s$ and $\Delta M_d$.

Subsequently $|V_{cb}|$ can be obtained from $\Delta M_s$ that depends only on $|V_{cb}|$ and very weakly on $\gamma$ and $\beta$ through $G(\beta ,\gamma )$ in (16) so that including also $\varepsilon _K$ and $\beta$ in this analysis the following values of the CKM parameters are found^6^ [31]

20 $$\begin{aligned} \boxed {\begin{aligned}|V_{cb}|&=42.6(4)\times 10^{-3}, \quad \gamma =64.6(16)^\circ ,\\ \beta&= 22.2(7)^\circ , \quad |V_{ub}|=3.72(11)\times 10^{-3}\,\end{aligned}} \end{aligned}$$

and consequently

21 $$\begin{aligned}{} & {} \boxed {\begin{aligned}&|V_{ts}|=41.9(4)\times 10^{-3}, \\&|V_{td}|=8.66(14)\times 10^{-3} ,\\&{{\textrm{Im}}}\lambda _t=1.43(5)\times 10^{-4} ,\end{aligned}} \end{aligned}$$

22 $$\begin{aligned}{} & {} \boxed {{\bar{\varrho }}=0.164(12),\quad {\bar{\eta }}=0.341(11) ,} \end{aligned}$$

where $$\lambda _t=V_{ts}^*V_{td}$$.

The values of $|V_{cb}|$ and $|V_{ub}|$ are in a very good agreement with the ones obtained in [14] from the $\Delta F=2$ processes alone. It should be noted that the determination of $\gamma$ in this manner, not provided in [14], is more accurate than its present determination from tree-level decays in (10). This very good agreement between the data and the SM for $\Delta F=2$ observables is an additional strong support for our strategy. Comparing with the (6) we observe that the determination of $|V_{cb}|$ in the global fit in [14] was indeed infected by NP because using the same hadronic input and restricting the analysis to $\Delta F=2$ processes these authors obtained practically the same results for $|V_{cb}|$ and $|V_{ub}|$ as in (20).

As emphasized in [31] and expressed here with the help of the formulae (13)–(15) and Fig. 1, this consistency in the $\Delta F=2$ sector is only found using the hadronic matrix elements with $2+1+1$ flavours from the lattice HPQCD collaboration [38]^7^ also used in [14]. These values are consistent with the inclusive determination of $|V_{cb}|$ in [32] and the exclusive ones of $|V_{ub}|$ from FLAG [4].

However, let me stress that the values in (20)–(22) are only a byproduct of our analysis. Except for $\beta$ obtained using SM expression in (11) I do not have to know other CKM parameters to obtain the SM predictions listed in Table 1 and in fact to obtain the predictions for all *K* and $B^0_{d,s}$ branching ratios within the SM.

#### UTfitter, CKMfitter and PDG 2022

It is instructive to compare our results for the CKM parameters with the most recent ones from the UTfitter [70],^8^ the CKMfitter and PDG22 [58]. These three groups perform global fits including $\Delta F=2$ observables, tree-level decays relevant for $|V_{cb}|$ and $|V_{ub}|$ determinations and dependently on the analysis some observables like the branching ratio for $B_s\rightarrow \mu ^+\mu ^-$ that still could be infected by NP. The same applies to the Cabibbo anomaly which has to be taken somehow into account in a global fit. The comparison in question is made in Table 2.

We observe that the values of ${\bar{\varrho }}$, ${\bar{\eta }}$, $|V_{td}|$ and $|V_{ub}|$ obtained by these three groups are in good agreement with ours, in particular the ones from the UTfitter. But the values of $|V_{cb}|$ and $|V_{ts}|$ are visibly lower with the ones from the UTfitter closer to ours than from the CKMfitter and PDG. This in turn implies the SM values of for all rare *K* and *B* decay branching ratios to be lower than ours. For $K^+\rightarrow \pi ^+\nu {\bar{\nu }}$ typically by $(5\pm 1)\%$ dependent on the fit. Presently these differences do not matter in view of large experimental errors but could be relevant in a few years from now.

The main origin of this difference is the inclusion of the tree-level determinations of $|V_{cb}|$ for which the tension between exclusive and inclusive determinations exists. It implies a lower value and larger error on this parameter and consequently when used in the calculations of branching ratios for theoretically clean decays a hadronic pollution of these decays. In our view the inclusion of the later determinations of $|V_{cb}|$ in a global CKM fit or any phenomenological analysis with the goal to predict SM branching ratios for rare *K* and *B* decays is not a good strategy at present. We think it should be avoided until these tensions are clarified.

Finally, our value for $\gamma$ is closer to its central value from the most recent LHCb measurement in (10) with the values from the CKMfitter and PDG by $1.7^\circ$ higher than the LHCb value and our only by $0.8^\circ$. It will be interesting to make such comparisons when the error on $\gamma$ from LHCb and Belle II will go down to $1^\circ$. As the theoretical error for the extraction of $\gamma$ from $B\rightarrow DK$ decays is tiny [71, 72], this determination will play a very important role for the tests of the SM and also of the $|V_{cb}|$ independent correlations between *K* and *B* decay branching ratios.

### SM predictions for $|V_{cb}|$-independent ratios

Among the 16 $|V_{cb}|$-independent ratios presented in [30] those that correlate *B* and *K* branching ratios depend on $\gamma$ and $\beta$. With the results in (20) at hand we can calculate them. The explicit expressions for these ratios as functions of $\beta$ and $\gamma$ are given in [30] and their compact collection can be found in [73]. Here we just list the final results using (20) which were not given there. Moreover in the case of the ratios $R_5$ and $R_7$ we use the most recent results for the formfactors entering ${\mathcal {B}}(B^+\rightarrow K^+\nu {\bar{\nu }})$ from the HPQCD collaboration [54–56].

23 $$\begin{aligned}{} & {} \boxed {\begin{aligned} R_0(\beta )&=\frac{{\mathcal {B}}(K^+\rightarrow \pi ^+\nu {\bar{\nu }})}{{\mathcal {B}}{(K_{L}\rightarrow \pi ^0\nu {\bar{\nu }})}^{0.7}}\\&={(2.03\pm 0.11)}\times {10}^{-3} , \end{aligned}} \end{aligned}$$

24 $$\begin{aligned}{} & {} \boxed {\begin{aligned}R_{\textrm{SL}}&=\frac{{{\mathcal {B}}}(K_S\rightarrow \mu ^+\mu ^-)_\textrm{SD}}{{{\mathcal {B}}}(K_{L}\rightarrow \pi ^0\nu {\bar{\nu }})}\\&=(6.29\pm 0.52)\times 10^{-3} ,\end{aligned}} \end{aligned}$$

25 $$\begin{aligned}{} & {} \boxed {\begin{aligned}R_1(\beta ,\gamma )&=\frac{{\mathcal {B}}(K^+\rightarrow \pi ^+\nu {\bar{\nu }})}{\left[ {\overline{{\mathcal {B}}}}(B_s\rightarrow \mu ^+\mu ^-)\right] ^{1.4}}\\&=53.69\pm 2.75 ,\end{aligned}} \end{aligned}$$

26 $$\begin{aligned}{} & {} \boxed {\begin{aligned} R_2(\beta ,\gamma )&=\frac{{\mathcal {B}}(K^+\rightarrow \pi ^+\nu {\bar{\nu }})}{\left[ {{\mathcal {B}}}(B_d\rightarrow \mu ^+\mu ^-)\right] ^{1.4}}\\&=(8.51\pm 0.47)\times 10^{-3} ,\end{aligned}} \end{aligned}$$

27 $$\begin{aligned}{} & {} \boxed {\begin{aligned}R_3(\beta ,\gamma )&=\frac{{\mathcal {B}}(K_{L}\rightarrow \pi ^0\nu {\bar{\nu }})}{\left[ {\overline{{\mathcal {B}}}}(B_s\rightarrow \mu ^+\mu ^-)\right] ^{2}}\\&=(2.08\pm 0.16)\times 10^6 .\end{aligned}} \end{aligned}$$

28 $$\begin{aligned}{} & {} \boxed {\begin{aligned}R_4(\beta ,\gamma )&=\frac{{\mathcal {B}}(K_{L}\rightarrow \pi ^0\nu {\bar{\nu }})}{\left[ {{\mathcal {B}}}(B_d\rightarrow \mu ^+\mu ^-)\right] ^{2}}\\&=(2.90\pm 0.24)\times 10^9 ,\end{aligned}} \end{aligned}$$

29 $$\begin{aligned}{} & {} \boxed {\begin{aligned}R_5(\beta ,\gamma )&=\frac{{\mathcal {B}}(K^+\rightarrow \pi ^+\nu {\bar{\nu }})}{\left[ {\mathcal {B}}(B^+\rightarrow K^+\nu {\bar{\nu }})\right] ^{1.4}}\\&=(1.90\pm 0.13)\times 10^{-3} ,\end{aligned}} \end{aligned}$$

30 $$\begin{aligned}{} & {} \boxed {\begin{aligned}R_6(\beta ,\gamma )&=\frac{{\mathcal {B}}(K^+\rightarrow \pi ^+\nu {\bar{\nu }})}{\left[ {\mathcal {B}}(B^0\rightarrow K^{0*}\nu {\bar{\nu }})\right] ^{1.4}}\\&=(8.82\pm 1.21)\times 10^{-4} .\end{aligned}} \end{aligned}$$

31 $$\begin{aligned}{} & {} \boxed {\begin{aligned}R_7&=\frac{{\mathcal {B}}(B^+\rightarrow K^+\nu {\bar{\nu }})}{{\overline{{\mathcal {B}}}}(B_s\rightarrow \mu ^+\mu ^-)}\\&= (1.50\pm 0.08)\times 10^{3} .\end{aligned}} \end{aligned}$$

32 $$\begin{aligned}{} & {} \boxed {\begin{aligned}R_8&=\frac{{\mathcal {B}}(B^0\rightarrow K^{*0}\nu {\bar{\nu }})}{{\overline{{\mathcal {B}}}}(B_s\rightarrow \mu ^+\mu ^-)}\\&=(2.62\pm 0.25)\times 10^{3} .\end{aligned}} \end{aligned}$$

One can check that the uncertainties in the ratios above are smaller than the ones one would find by calculating them by means of the results in Table 1 because some uncertainties cancel in the ratio when they are calculated directly using the expressions in [30, 31].

The ratios $R_{9}$ and $R_{10}$ involve only $|\varepsilon _K|$ and $\Delta M_{s,d}$ which were used in the rapid test and in the determination of the CKM parameters from (2) so that we can skip them here. Presently, most interesting are the ratios in (7)–(9 for which we find

33 $$\begin{aligned}{} & {} \boxed {\begin{aligned}R_s&=\frac{{\mathcal {B}}(B_s\rightarrow \mu ^+\mu ^-)}{\Delta M_s}\\&= (2.13\pm 0.07)\times 10^{-10}\text {ps} ,\end{aligned}} \end{aligned}$$

34 $$\begin{aligned}{} & {} \boxed {\begin{aligned}R_d&=\frac{{\mathcal {B}}(B_d\rightarrow \mu ^+\mu ^-)}{\Delta M_d}\\&=(2.02\pm 0.08)\times 10^{-10}\text {ps} ,\end{aligned}} \end{aligned}$$

35 $$\begin{aligned}{} & {} \boxed {\begin{aligned}R_{11}(\beta ,\gamma )&=\frac{{\mathcal {B}}(K^+\rightarrow \pi ^+\nu {\bar{\nu }})}{|\varepsilon _K|^{0.82}}\\&=(1.31\pm 0.06)\times 10^{-8} , \end{aligned}} \end{aligned}$$

36 $$\begin{aligned}{} & {} \boxed {\begin{aligned}R_{12}(\beta ,\gamma )&=\frac{{\mathcal {B}}(K_{L}\rightarrow \pi ^0\nu {\bar{\nu }})}{|\varepsilon _K|^{1.18}}\\&=(3.87\pm 0.13)\times 10^{-8} . \end{aligned}} \end{aligned}$$

## SM predictions for $H_1\rightarrow H_2\mu ^+\mu ^-$ branching ratios

The semi-leptonic transitions $b\rightarrow s\ell ^+\ell ^-$ have been left out in [30, 31] because of larger hadronic uncertainties than is the case of decays listed in Table 1. However, in fact having the result for $|V_{ts}|$ in (21) we can next calculate all branching ratios involved in the *B*-physics anomalies. To this end we use a very useful formula [14]

37 $$\begin{aligned} {\mathcal {B}}(H_1\rightarrow H_2\mu ^+\mu ^-)_\text {SM}^{[q^2_{\text {min}},q^2_{\text {max}}]}=|V_{ts}|^2 a_{H_1\rightarrow H_2}^{[q^2_{\text {min}},q^2_{\text {max}}]}, \end{aligned}$$

where the superscript $[q^2_{\text {min}},q^2_{\text {max}}]$ indicates $q^2$ bin. For each decay mode the authors of [14] calculated the numerical coefficients in front of $|V_{ts}|^2$ for one broad $q^2$ bin below the narrow charmonium resonances and one broad bin above. For the numerical coefficients in (37) they find [14]

38 $$\begin{aligned} a_{B^+\rightarrow K^+}^{[1.1,6]}= & {} (1.00\pm 0.16)\times 10^{-4}, \nonumber \\ a_{B^+\rightarrow K^+}^{[15,22]}= & {} (0.61\pm 0.06)\times 10^{-4}, \end{aligned}$$

39 $$\begin{aligned} a_{B^0\rightarrow K^{*0}}^{[1.1,6]}= & {} (1.36\pm 0.16)\times 10^{-4}, \nonumber \\ a_{B^0\rightarrow K^{*0}}^{[15,19]}= & {} (1.39\pm 0.15)\times 10^{-4}, \end{aligned}$$

40 $$\begin{aligned} a_{B_s\rightarrow \phi }^{[1.1,6]}= & {} (1.54\pm 0.14)\times 10^{-4}, \nonumber \\ a_{B_s\rightarrow \phi }^{[15,19]}= & {} (1.30\pm 0.12)\times 10^{-4} . \end{aligned}$$

41 $$\begin{aligned} a_{\Lambda _b\rightarrow \Lambda }^{[1.1,6]}= & {} (0.30\pm 0.16)\times 10^{-4},\nonumber \\ a_{\Lambda _b\rightarrow \Lambda }^{[15,20]}= & {} (2.07\pm 0.21)\times 10^{-4}. \end{aligned}$$

These results are based on [26, 74, 75]. However, recently new results from HPQCD collaboration with $2+1+1$ flavours [54–56] for $B^+\rightarrow K^+$ formfactors became available from which we extract

42 $$\begin{aligned} a_{B^+\rightarrow K^+}^{[1.1,6]}= & {} (1.18\pm 0.08)\times 10^{-4}, \nonumber \\ a_{B^+\rightarrow K^+}^{[15,22]}= & {} (0.72\pm 0.05)\times 10^{-4}, \nonumber \\{} & {} (\text {HPQCD22}). \end{aligned}$$

We will use these results instead of (38) in what follows.

Using then these coefficients together with $|V_{ts}|$ in (21) we obtain the results for various branching ratios listed in Table 3. We compare them with the data and list the pulls in the last column. While some pulls are in the ballpark of $(2-3)\sigma$, we find a $-4.8\sigma$ anomaly in $B_s\rightarrow \phi \mu ^+\mu ^-$ in the lower $q^2$ bin. This finding agrees with the one of [14]. Similarly a large pull of $-4.7\sigma$ in the low $q^2$ bin in $B^+\rightarrow K^+\mu ^+\mu ^-$ has been found recently by HPQCD colaboration [55]. With our CKM parameters it is further increased to $-5.1\sigma$.^9^ These appear to be the largest anomalies in single branching ratios.

It should be noted that for all branching ratios in Table 3 one can construct, with the help of $\Delta M_s$, the CKM independent ratios as in the previous section. Here we just present the results for the two among them in the low $q^2$ bin that exhibit the largest pulls mentioned above. We find

43 $$\begin{aligned} \boxed {\begin{aligned}R_{13}&=\frac{{\mathcal {B}}(B^+\rightarrow K^+\mu ^+\mu ^-)}{\Delta M_s}\\&=(1.167\pm 0.079)\times 10^{-8} \\&\quad \left[ \frac{256.1\text {MeV}}{\sqrt{{\hat{B}}_{B_s}}F_{B_s}}\right] ^2\,\text {ps} ,\quad [1.1,6] \end{aligned}} \end{aligned}$$

and

44 $$\begin{aligned} \boxed {\begin{aligned}R_{14}&=\frac{{\mathcal {B}}(B^+\rightarrow \phi \mu ^+\mu ^-)}{\Delta M_s}\\&=(1.523\pm 0.138)\times 10^{-8} \\&\quad \left[ \frac{256.1\text {MeV}}{\sqrt{{\hat{B}}_{B_s}}F_{B_s}}\right] ^2\,\text {ps} ,\quad [1.1,6] .\end{aligned}} \end{aligned}$$

Including the uncertainty in $\sqrt{{\hat{B}}_{B_s}}F_{B_s}$ we find

45 $$\begin{aligned}{} & {} \boxed {\begin{aligned}R_{13}&= (1.167\pm 0.095)\times 10^{-8}\,\text {ps},\quad \\ R_{13}^{\text {EXP}}&= (0.668\pm 0.038)\times 10^{-8}\,\text {ps} ,\quad [1.1,6] ,\end{aligned}} \end{aligned}$$

46 $$\begin{aligned}{} & {} \boxed {\begin{aligned}R_{14}&= (1.523\pm 0.154)\times 10^{-8}\,\text {ps},\quad \\ R_{14}^{\text {EXP}}&= (0.794\pm 0.056)\times 10^{-8}\,\text {ps},\quad [1.1,6]\end{aligned}} \end{aligned}$$

and the pulls $-4.9\sigma$ and $-4.5\sigma$, respectively. The reduction of the pulls relative to the ones for branching ratios in Table 3 originates in the larger error from the hadronic uncertainty in $\sqrt{{\hat{B}}_{B_s}}F_{B_s}$ than the uncertainty in $|V_{ts}|$ obtained from the $\Delta F=2$ fit that involves also $\Delta M_d$, $|\varepsilon _K|$ and $S_{\psi K_S}$. But the advantage over the branching ratios themselves is that these ratios are free from any CKM dependence.

Importantly, the experimental branching ratios are for most of the branching ratios in Table 3 below the SM predictions which expresses the anomalies widely discussed in the literature. It should also be emphasized that studying various differential distributions, various asymmeteries $S_i$ and $A_i$ as proposed in [76] or $P_i(P_i^\prime )$ variables proposed in [77] that suffer from smaller hadronic and parameteric uncertainties than branching ratios themselves the pulls in $B\rightarrow K(K^*)\mu ^+\mu ^-$ could turn out to be larger. Yet, just testing the branching ratios themselves is much simpler and can give already some indications on the presence of NP.

## SM predictions for $b\rightarrow s\nu {\bar{\nu }}$ transitions

Several SM branching ratios for *B* decays with neutrino pair in the final state beyond those discussed by us above have been calculated in [78] with a much lower value of $|V_{ts}|=39.7\times 10^{-3}$ than used by us.^10^ We present in Table 4 the corresponding results with our value of $|V_{ts}|$ in (21). They are typicaly by $11\%$ higher than the ones in [78]. The interest in the *B* decays with neutrino pair in the final was already significant for years^11^ but it increased recently due to the BELLE II experiment [82] as seen in [46, 78, 83–87].

## Direct route to SM predictions for $H_1\rightarrow H_2\mu ^+\mu ^-$ branching ratios and $b\rightarrow s\nu {\bar{\nu }}$

It should be stressed that the predictions in Sects. 5 and  6 go beyond the main strategy of removing CKM parameters from the analyses and we report here how our results in the previous two sections would change if we eliminated $|V_{ts}|$ with the help of $\Delta M_s$ and setting its value to the experimental one. This procedure is a bit safer as the results are expected to be more stable under future modifications of $|V_{ts}|$ due to possible changes in non-perturbative parameters in the $\Delta F=2$ system beyond those relevant for $\Delta M_s$. Basically the present uncertainty from $|V_{ts}|^2$ of $1.9\%$ obtained from the full $\Delta F=2$ fit increases to $4.4\%$. But as the uncertainties in the formfactors have presently a significantly larger impact on the error in the final preditions these changes are small. In particular the central values are not modified because, as seen in Fig. 1, $\Delta M_s$ being only very weakly dependent on $\gamma$ plays an important role in the determination of $|V_{cb}|$ in the full $\Delta F=2$ fit. We just quote a few examples in the modifications of the resulting errors:

47 $$\begin{aligned}{} & {} B^+\rightarrow K^+\mu ^+\mu ^-~ ([1.1,6]): (2.07\pm 0.16)\cdot 10^{-7} \nonumber \\{} & {} \quad \rightarrow (2.07\pm 0.18)\cdot 10^{-7} , \end{aligned}$$

48 $$\begin{aligned}{} & {} B^0\rightarrow K^{*0}\mu ^+\mu ^-~([1.1,6]): (2.39\pm 0.28)\cdot 10^{-7} \nonumber \\{} & {} \quad \rightarrow (2.39\pm 0.30)\cdot 10^{-7} , \end{aligned}$$

49 $$\begin{aligned}{} & {} B_d^0\rightarrow K^{*0}\mu ^+\mu ^-~ ([15,19]): (2.44\pm 0.26)\cdot 10^{-7} \nonumber \\{} & {} \quad \rightarrow (2.44\pm 0.28)\cdot 10^{-7} , \end{aligned}$$

50 $$\begin{aligned}{} & {} B_s\rightarrow \phi \mu ^+\mu ^-~([1.1,6]): (2.70\pm 0.25)\cdot 10^{-7}\nonumber \\{} & {} \quad \rightarrow (2.70\pm 0.27)\cdot 10^{-7} , \end{aligned}$$

51 $$\begin{aligned}{} & {} B_s\rightarrow \phi \mu ^+\mu ^-~([15,19]): (2.28\pm 0.21)\cdot 10^{-7} \nonumber \\{} & {} \quad \rightarrow (2.28\pm 0.23)\cdot 10^{-7} . \end{aligned}$$

In the case of final states with $\nu {\bar{\nu }}$ these changes are described in Table 4.

## Exclusive and hybrid scenarios

But what if one day experts agree on the basis of tree-level decays that the values of the CKM parameters differ from those that are listed in (20). For instance one could consider, as done in [31], the following two well defined scenarios based on tree-level decays. First the EXCLUSIVE one

52 $$\begin{aligned}{} & {} |V_{cb}|=39.21(62)\times 10^{-3},\quad |V_{ub}|=3.61(13)\times 10^{-3},\nonumber \\{} & {} \quad (\textrm{EXCLUSIVE}) \end{aligned}$$

that summarize preliminary results from FLAG2022 and the HYBRID one in which the value for $|V_{cb}|$ is the inclusive one from [32] and the exclusive one for $|V_{ub}|$ as above:

53 $$\begin{aligned}{} & {} |V_{cb}|=42.16(50)\times 10^{-3},\quad |V_{ub}|=3.61(13)\times 10^{-3},\nonumber \\{} & {} \quad {(\mathrm HYBRID)}. \end{aligned}$$

The important point to be stressed here is the following one. The SM predictions for those $|V_{cb}|$ independent ratios, defined in [30] and evaluated in Sect. 4.4 that are independent of all CKM parameters, will be modified in the future only by changes in hadronic parameters. In the ratios involving *K* decays the value of $\beta$ matters and could modify the ratios in addition in the future. However, as seen in (8) and (9), for $R_{11}$ and $R_{12}$ the $\gamma$ dependence is negligible. Other ratios can depend significantly on $\gamma$ and $\beta$ and this dependence is exhibited in numerous plots in [30].

But the values of the branching ratios and also of $\Delta M_s$, $\Delta M_d$ and $\varepsilon _K$ will change, in particular by much in the exclusive scenario. However, it will happen in a correlated manner with correlations simply described by the $|V_{cb}|$-independent ratios.

In particular, as analysed in detail in [31], in the exclusive scenario significant anomalies in $\Delta M_s$, $\Delta M_d$ and $\varepsilon _K$ will be found, while several ones in *B* decays will be removed or decreased. For instance all branching ratios in Tables 3 and 4 will be suppressed by a factor 0.847 reducing significantly the present anomalies and in the case of the $B_s\rightarrow \mu ^+\mu ^-$ decay removing it completely. But the room for NP opened in the $\Delta F=2$ sector will significantly weaken the constraints on NP from this sector. As seen in [31], in the hybrid scenario the results do not differ by much from the ones presented here but have larger errors dominantly due to larger error on $\gamma$ than in (20).

## Searching for footprints of NP beyond the SM

Having the results from our strategy at hand, the simplest route to find out whether there is some NP, once the experimental values of many branching ratios will be known, is in my view the following one:

**Step 1:**

Comparison of CKM-independent ratios like (7) with experiment. In the case of $R_s$ there was already a sign of NP. The SM prediction for $R_s$ and the resulting SM prediction for $B_s\rightarrow \mu ^+\mu ^-$ branching ratio differed by $2.7\sigma$ from the data. However, this difference has been reduced by much due to the recent CMS result. Once $B_d\rightarrow \mu ^+\mu ^-$ branching ratio will be measured, similar test will be possible for $R_d$ and other decays like $B\rightarrow K(K^*)\nu {\bar{\nu }}$. Even more interesting are the pulls in the low $q^2$ bin in the ratios $R_{13}$ and $R_{14}$ involving $B^+\rightarrow K^+\mu ^+\mu ^-$ ($-4.9\sigma$) and $B_s\rightarrow \phi \mu ^+\mu ^-$ ($-4.5\sigma$), respectively.

When the branching ratios for $K^+\rightarrow \pi ^+\nu {\bar{\nu }}$, $K_{L}\rightarrow \pi ^0\nu {\bar{\nu }}$ and other rare *K* decays will be measured, SM predictions will be tested through ratios like $R_{11}$ and $R_{12}$ that depend practically only on $\beta$.

It should be stressed that all these ratios do not involve the assumption of the absence of NP in $\Delta F=2$ observables and in the case of the sign of NP in the ratio it could come from the $\Delta F=1$ observable or $\Delta F=2$ observable or even both.

**Step 2:**

Once the rapid test in Sect. 4.2 is found to be negative one can set the $\Delta F=2$ observables to their experimental values. This allows to predict the branching ratios either by means of the $|V_{cb}|$-independent ratios or just using the CKM parameters determined exclusively from $\Delta F=2$ observables. The results for the branching ratios are collected in Tables 1, 3 and 4. Similarly, one can calculate those $|V_{cb}|$-independent ratios of [30] that depend on $\beta$ and $\gamma$. The results are given in (23)–(36) and (43)–(46).

Following these steps, future measurements of all branching ratios calculated in the present paper will hopefully tell us what is the pattern of deviations from their SM predictions allowing us to select some favourite BSM models. Indeed in this context various $|V_{cb}|$ independent ratios of branching ratios considered by us, both independent of $\beta$ and $\gamma$ and dependent on them and calculated by us in Sect. 4.4 will provide a good test of the SM. Similarly $|V_{cb}|-\gamma$ plots [30, 31, 68] will play an important role, in particular if $\beta$ and $\gamma$ will be determined in tree-level non-leptonic *B* decays that are likely to receive only very small NP contributions. However, this may still take some time. Then also the comparison with the values in (20) will be possible. Moreover, beyond the SM the ratios $R_i$ will depend on $|V_{cb}|$ so that its value will be necessary for the study of NP contributions. Therefore, it is very important that this direct route to $|V_{cb}|$ through trevel decays is continued with all technology we have to our disposal.

**Table 5:** *Values of the experimental and theoretical quantities used as input parameters. For future updates see FLAG* [4], *PDG* [48] *and HFLAV* [44, 113]

$m_{B_s} = 5366.8(2)\text {MeV}$ [48]	$m_{B_d}=5279.58(17)\text {MeV}$ [48]
$\Delta M_s = 17.749(20) \,\text {ps}^{-1}$ [48]	$\Delta M_d = 0.5065(19) \,\text {ps}^{-1}$ [48]
$\Delta M_K = 0.005292(9) \,\text {ps}^{-1}$ [48]	$m_{K^0}=497.61(1)\text {MeV}$ [48]
$S_{\psi K_S}= 0.699(17)$ [48]	$F_K=155.7(3)\text {MeV}$ [4]
$|V_{us}|=0.2253(8)$ [48]	$|\epsilon _K|= 2.228(11)\cdot 10^{-3}$ [48]
$F_{B_s}$ = $230.3(1.3)\text {MeV}$ [4]	$F_{B_d}$ = $190.0(1.3)\text {MeV}$ [108]
$F_{B_s} \sqrt{{\hat{B}}_s}=256.1(5.7) \text {MeV}$ [38]	$F_{B_d} \sqrt{{\hat{B}}_d}=210.6(5.5) \text {MeV}$ [38]
${\hat{B}}_s=1.232(53)$ [38]	${\hat{B}}_d=1.222(61)$ [38]
$m_t(m_t)=162.83(67)\,\text {GeV}$ [109]	$m_c(m_c)=1.279(13)\,\text {GeV}$
$S_{tt}(x_t)=2.303$	$S_{ut}(x_c,x_t)=-1.983\times 10^{-3}$
$\eta _{tt}=0.55(2)$ [21]	$\eta _{ut}= 0.402(5)$ [21]
$\kappa _\varepsilon = 0.94(2)$ [110]	$\eta _B=0.55(1)$ [15, 111]
$\tau _{B_s}= 1.515(4)\,\text {ps}$ [112]	$\tau _{B_d}= 1.519(4)\,\text {ps}$ [112]

## Conclusions and outlook

We have pointed out that the most straightforward method for obtaining SM predictions for rare *K* and *B* decays is to study those SM correlations between the branching ratios and $\Delta F=2$ observables that do not depend or depend minimally on the CKM parameters. The standard method is to determine the latter first through global fits and subsequently insert the resulting values into SM formulae. In view of the mounting evidence for NP in semi-leptonic *B* decays the resulting values of the CKM parameters are likely to be infected by NP if such decays are included in a global fit. Inserting them in the SM expressions for rare decays in question will obviously not provide genuine SM predictions for their branching ratios.

The determination of the CKM parameters *exclusively* from tree-level decays could in principle reduce the dependence of CKM parameters on NP^12^ and the prospects of their determination in the coming years are good [99]. However, the present tensions between inclusive and exclusive determination of $|V_{cb}|$ is a stumbling block on this route to SM predictions of branching ratios that are very sensitive to $|V_{cb}|$ [30]. As demonstrated in [31] going this route using the exclusive determination of $|V_{cb}|$ would result in very different predictions than obtained by using the corresponding inclusive route. The recent analysis in [29] demonstrates this problem as well.

As proposed very recently in [100] the sum $|V_{td}|^2+|V_{ts}|^2$ could also be accessed through CKM suppressed top decays at the LHC. We note that this would provide another route to $|V_{cb}|$ through

54 $$\begin{aligned} |V_{td}|^2+|V_{ts}|^2= & {} |V_{cb}|^2[G^2(\beta ,\gamma )+\lambda ^2 R_t^2],\nonumber \\ R_t= & {} \frac{\sin \gamma }{\sin (\beta +\gamma )} , \end{aligned}$$

where $G(\beta ,\gamma )$ is given in (16) with $\beta$ and $\gamma$ determined through tree-level non-leptonic *B* decays. This would avoid the use of presently controversial value of $|V_{cb}|$ from tree-level semi-leptonic *B* decays. This would also provide another test of our values of the CKM parameters. Using them we find

55 $$\begin{aligned} |V_{td}|^2+|V_{ts}|^2=42.8(4)\times 10^{-3} . \end{aligned}$$

It should be emphasized that to obtain precise SM predictions like the ones in Table 1 it is crucial to choose the proper pairs of observables. For instance combining $K^+\rightarrow \pi ^+\nu {\bar{\nu }}$ with $\Delta M_s$ or $B_s\rightarrow \mu ^+\mu ^-$ with $\varepsilon _K$ would not allow us precise predictions for $K^+\rightarrow \pi ^+\nu {\bar{\nu }}$ and $B_s\rightarrow \mu ^+\mu ^-$ even after the elimination of the $|V_{cb}|$ because of the left-over $\gamma$ dependence in both cases. Moreover selecting a subset of optimal observables for a given SM prediction with the goal of removing the CKM dependence avoids the assumption of the absence of NP in other observables that enter necessarily a global fit.

It is known from numerous studies that NP could have significant impact on $\Delta F=2$ observables, in particular in the presence of left-right operators which have enhanced hadronic matrix elements and their contributions to $\Delta F=2$ processes are additionally enhanced through QCD renormalization group effects. One could then ask the question how in the presence of significant NP contributions to semi-leptonic decays one could avoid large contributions to $\Delta F=2$ observables. Some answers are given in the 4321 model [101, 102] and in a number of analyses by Isidori’s group [103–107] in which a specific flavour structure allows to suppress the contributions to $\Delta F=2$ processes from the leptoquark $U_1$, heavy $Z^\prime$, $G^\prime$ and vector-like fermions while allowing for their sizeable contributions to semileptonic decays. Yet, the fact that the SM performs so well in the $\Delta F=2$ sector when the HPQCD results [38] are used puts even stronger constraints on NP model constructions than in the past. Therefore it is crucial that other LQCD collaborations perform $2+1+1$ calculations of $\Delta F=2$ hadronic matrix elements.

In the spirit of the last word in the title of our paper it will be of interest to see one day whether the archipelago of $\Delta F=2$ observables will be as little infected by NP as has been the Galapagos archipelago by Covid-19 and other pandemics in the past. The expressions in Sect. 4.2 provide a *rapid test* in this context. This test will improve with the reduction of the uncertainties in ${\hat{B}}_K$, $\sqrt{{\hat{B}}_{B_d}}F_{B_d}$, $\sqrt{{\hat{B}}_{B_s}}F_{B_s}$ and $\beta$.

However, even if this test would fail and NP would infect $\Delta F=2$ observables, the $|V_{cb}|$ independent ratios introduced in [30, 36], in particular those free of the CKM parameters, will offer excellent tests of the SM dynamics. Such tests will be truly powerful when the uncertainties on $\gamma$ and $\beta$ from tree-level decays will be reduced in the coming years.

We are looking forward to the days on which numerous results presented in Tables 1, 2, 3 and 4, in the formulae (23)–(36) and (43)–(46) will be compared with improved experimental data. In particular it is of great interest to see whether the anomalies in the low $q^2$ bin in $B^+\rightarrow K^+\mu ^+\mu ^-$ ($5.1\sigma$) and $B_s\rightarrow \phi \mu ^+\mu ^-$ ($4.8\sigma$) will remain even if the violation of the lepton flavour universality in semi-leptonic decays would disappear.

## Data Availability

This manuscript has no associated data or the data will not be deposited. [Authors’ comment: It is a theory paper and there are no data attached to it nor codes etc.]

## Footnotes

1 This choice is more useful than the one in which $\beta$ is replaced by $|V_{ub}|$, allowing for much simpler CKM factors than in the latter case used e.g. recently in [14].

2 This point has been already made in a short note by the present author [28] and very recently in [29] but the solution to this problem suggested in the latter paper is drastically different from the one proposed here that is based on [30, 31]. We will comment on it below.

3 The exclusive value for $|V_{cb}|$ should be considered as preliminary.

4 This question applies also to global fits related to the tests of lepton flavour universality violation in which the CKM input only from tree-level decays is used. See [33] and references therein.

5 The nominal value of $\gamma$ in these expression as used in [30, 31] differs from $\gamma =64.6^\circ$ used by us in subsequent papers but inserting the latter has practically no impact on the numerical coefficients in these ratios.

6 $|V_{us}|$ is given in Table 5.

7 Similar results for $\Delta M_d$ and $\Delta M_s$ hadronic matrix elements have been obtained within the HQET sum rules in [6, 69], respectively.

8 I thank Luca Silvestrini for discussion of these most recent results.

9 We thank Will Parrott from the HPQCD collaboration for confirming this result.

10 For $B_s\rightarrow \phi \nu {\bar{\nu }}$ ref. [79] confirms the results of [78] using practically the same value of $|V_{ts}|$.

11 See [80, 81] and the references therein.

12 Nonetheless, NP can also affect these decays as stressed in [94–98].
